# Effect of Elevated Temperature on the Compressive Strength and Durability Properties of Crumb Rubber Engineered Cementitious Composite

**DOI:** 10.3390/ma13163516

**Published:** 2020-08-10

**Authors:** Bashar S. Mohammed, Lee Yin Yen, Sani Haruna, Michael Lim Seng Huat, Isyaka Abdulkadir, Amin Al-Fakih, M. S. Liew, Noor Amila Wan Abdullah Zawawi

**Affiliations:** 1 Department of Civil and Environmental Engineering, Universiti Teknologi PETRONAS, Bandar Seri Iskandar 32610, Malaysia; bashar.mohammed@utp.edu.my (B.S.M.); leeyinyen_19801@utp.edu.my (L.Y.Y.); limsenghuat_20990@utp.edu.my (M.L.S.H.); isyaka_18000638@utp.edu.my (I.A.); amin.ali_g03663@utp.edu.my (A.A.-F.); shahir_liew@utp.edu.my (M.S.L.); amilawa@utp.edu.my (N.A.W.A.Z.); 2 Department of Civil Engineering, Bayero University Kano, 3011 Kano, Nigeria

**Keywords:** engineered cementitious composite, crumb rubber, residual compressive strength, elevated temperature, weight loss

## Abstract

This paper reports the findings of the effect of elevated temperature on the compressive strength and durability properties of crumb rubber engineered cementitious composite (CR-ECC). The CR-ECC has been tested for its compressive strength and chemical resistance test against acid and sulphate attack. Different proportions of crumb rubber (CR) in partial replacement to the fine aggregate and polyvinyl alcohol (PVA) fiber have been utilized from 0 to 5% and 0 to 2%. The experiments were designed based on a central composite design (CCD) technique of response surface methodology (RSM). After 28 days curing, the samples were preconditioned and exposed to high temperatures of 100 °C, 200 °C, 300 °C, 400 °C, 500 °C, 600 °C, 700 °C, 800 °C, 900 °C, and 1000 °C for one hour. Although the residual compressive strength of CR-ECC was negatively affected by elevated temperature, no explosive spalling was noticed for all mixes, even at 1000 °C. Results indicated that CR-ECC experiences slight weight gain and a reduction in strength when exposed to the acidic environment. Due to the reduced permeability, CR-ECC experienced less effect when in sulphate environment. The response models were generated and validated by analysis of variance (ANOVA). The difference between adjusted R-squared and predicted R-squared values for each model was less than 0.2, and they possess at least a 95% level of confidence.

## 1. Introduction

The ever-increasing environmental problem caused by waste tire disposal has made the use of crumb rubber in engineered cementitious composite (ECC) one of the viable alternatives to eradicating the menace. The issue of waste tire disposal is one of the significant environmental challenges facing cities around the world. It is estimated that about one billion waste tires are generated annually, and this figure is going to increase as the growth in population and advancement in technology gives rise to the production of more cars [1]. The alarming rate by which waste tire is generated is posing a threat to human health and the safety of the environment. This is because the dumpsite becomes breeding grounds for pests and vermin [2]. To address this global problem, various research is conducted to examine numerous possible applications of recycled tire rubber [3,4,5]. One of the ways to find a solution is in the utilization of the waste tire rubber as a sustainable construction material. To this end, numerous researches using crumb rubber from the waste tire as a partial replacement for fine aggregate have been carried out [6,7,8,9,10]. Results indicated that incorporating CR in concrete led to an increase in energy-absorbing characteristics, increased ductility, reduced brittle failure, and freeze-thaw resistance [11]. In this study, an engineered cementitious composite containing crumb rubber (CR-ECC) in various percentages will be used with the view to find its behavior at elevated temperatures. There are relatively few works on the use of CR in engineered cementitious composites (ECCs).

Engineered cementitious composite (ECC) is a type of high-performance fiber-reinforced cementitious composite (HPFRCC) that is designed based on the theories of micromechanics resulting in its exceptional ductility [12,13,14]. In ECC, the fiber volume fraction (usually polyvinyl alcohol fiber), is kept at an optimized level of no more than 2% [2]. ECC has many advantages over other types of concretes, one of which is its ability to illustrate strain-hardening characteristics rather than tension softening. This is due to its high ductility under uniaxial tensile loads. It has a tensile strain capacity of 3–5%, and that is about 300–500 times than the tensile capacity of conventional concrete and fiber reinforced concrete [15,16]. Another advantage of ECC over ordinary types of concretes is its inherently tight crack width characteristic of less than 100 µm. The tight crack widths that develop during ECC’s strain hardening process makes it very durable under different adverse conditions because of the low permeability coefficient and chloride diffusion property, unlike in ordinary concretes where localized cracks exhibit continuous widening [13,17]. These excellent properties, in addition to its versatility, are the reasons behind ECC’s popularity over the last few years [12].

Despite its numerous advantages, ECC like other high strength concretes, has one major disadvantage, which is its explosive spalling tendency [18]. Explosive spalling refers to the sudden and intense breaking away of the surface layer of concrete at elevated temperatures [19]. Most of the relevant available literature focused on the effect of elevated temperature on ECC incorporating PVA fibers. What is most common in the findings of all research reviewed on ECC with PVA fibers was that the presence of the fibers reduces the explosive spalling tendencies of the ECC at elevated temperatures up to 800 °C. That behavior is attributed to the change in the pore structure by the melting of the PVA fibers at a temperature of 230 °C. The pores created serve as escape routes for the water vapor within the dense ECC matrix, thereby eliminating the chance of the explosive spalling due to the buildup of the vapor pressure [16]. Aside from that, numerous studies on the behavior of ECC at elevated temperatures have been conducted with interesting findings. Yu et al. [13] assessed the mechanical performance of ECC with high volume fly ash at sub-elevated temperatures of 50 °C, 100 °C and 200 °C and concluded that the ECC’s compressive strength was unaffected at 50 °C and 100 °C heating, however, it dropped when exposed to a temperature of 200. At 200 °C, it was noted that the typical behaviors of the ECC, such as strain hardening, and tight crack width were conserved even after heating for one hour. The high-volume fly ash ECC showed a better tensile ductility and strain hardening characteristics at 50 to 100 °C temperature exposure than specimens left at ambient temperature, although there was a small decrease at 200 °C [13]. Similarly, it was discovered that the ECC lost its tensile strain-hardening capacity at a temperature beyond 200 °C, and its tensile strength reduced by approximately 40% [19]. Interestingly, however, the compressive strength of the ECC was found to be unaffected even after subjecting it to 600 °C for 6 h, which is in sharp contrast to the findings of [15], that indicated a reduction in compressive strength at a temperature above 600 °C.

Durability studies on rubberized ECC have shown that the presence of CR reduces the origination and propagation of cracks which in turn will lead to reduced ingress of aggressive media [20]. Liu et al. [21] reported that CC remained durable even after 200 days of exposure to aggressive environments. The result of research conducted by Assas [22] showed that incorporating CR up to 15% led to the concrete having higher resistance to sulphate attack better than the control concrete having no CR. However, despite this research on the durability of ECC, the literature on the optimization and durability performance of CR-CC in sulphate and an acidic environment is almost non-existent.

From the foregoing, it can be understood that research focused mainly on normal ECC, high volume fly ash ECC, hybrid fiber (PVA and steel fiber) ECC, etc. There is limited work on crumb rubber ECC subjected to high temperatures. That is because of the lack of literature on the behavior of CR-ECC at elevated temperatures, and that is the focus of this paper. Furthermore, this research will go beyond the usual 600–800 °C maximum elevated temperature limit, as seen in most of the previous research, to 1000 °C. In this research, Response surface methodology (RSM) will be used in the ECC design, modeling, and in the determination of the optimized levels of the variables that will give the most desirable or optimum response. Response surface methodology (RSM) is a potent experimental design method for the modeling and analysis of problems in which a response (output parameter) of interest is influenced by several independent variables (input parameters). The use of RSM in the field of ECC design and optimization is not new because of its efficiency and advantages [15,23].

## 2. Materials and Methods

### 2.1. Materials

**Figure 1:**
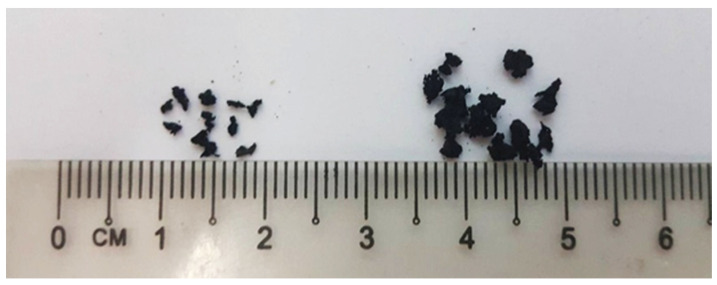
Crumb rubber with size range of 0.6 mm to 2.36 mm.

The materials used in preparing the crumb rubber engineered cementitious composite (CR-ECC) mixes were: Type 1 ordinary Portland cement (OPC) conforming to the specifications of ASTM C150 [24]. Class F fly ash (FA) conforming to the specifications of ASTM C618 [25]. The oxide composition, specific gravity, and Blaine fineness of the OPC and fly ash are shown in Table 1. River sand passing No. 4 sieve (less than 4.75 mm in size) was used as the fine aggregate. Poly vinyl alcohol (PVA) fiber produced by Kuraray Japan was used. The surface of the fiber was coated with 1.2% oil by weight to control the fiber/matrix interfacial properties. Properties of the fiber are shown in Table 2. Crumb rubber is obtained from waste tires. The process involves using a shredder to cut the waste tire rubber into tiny pieces. For this research, the CR used has a size range between 600 µm–2.36 mm as shown in Figure 1. Superplasticizer used for the mixture was a modified polycarboxylate-based high range water reducer with a pH value of 6.2, specific gravity of 1.08, and free chloride content of 0.1%. The superplasticizer dose added to the mixture was 0.99% by weight of cementitious materials.

### 2.2. Development of Predictive Models Using Response Surface Methodology

Response surface methodology is a set of mathematical and statistical tools used for building an analytical model in which a response (an output) is related to many input variables which are independent [26]. The method can be used in assessing the effects of each of the parameters and the relationship between them on the response [27,28,29]. In this research, RSM was used to develop the predictive models and to optimize the compressive strength (as the response) and the amount of CR and the elevated temperature (as the independent variables) in the RC-ECC. The Design of Experiment (DOE) software was used for the RSM analysis. The most commonly used design methods in the field of civil engineering are central composite design (CCD) and Box–Behnken design (BBD). Design expert software has been utilized for developing the mix designs and optimizations. The optimization process involves three major steps: (1) conducting the statistically designed experimental work, (2) predicting the coefficients in a mathematical model, and (3) predicting the model’s response and verifying its adequacy [30,31]. The developed statistical models can either take the pattern of linear or higher-degree polynomials between the independent parameters and their responses. The linear equation is presented by a first-order equation as shown in Equation (1). while the polynomials models are presented in Equation (2). The y symbol represents the response models, *x_i_* and *x_j_* are the coded value of the input variables, *i* and *j* are linear and quadratic coefficients, β_o_ represent the intercept on the y-axis, k represents the number of the independent variables used in the model, and ε is the error in the model developed [32].

(1) $$y=\beta o+\beta_{1}x_{1}+\beta_{2}x_{2}+\cdots +\beta_{n}x_{n}+\varepsilon$$

(2) $$y=\beta o+\sum_{\dot{i}=1}^{k}\beta_{i}x_{i}+\sum_{i=1}^{k}\beta_{ii}X_{j^{2}}+\sum_{j=2}^{k}\sum_{\dot{i}=1}^{j=1}\beta_{ij}\;X_{i}X_{j}+\varepsilon$$

Using a *p*-value of 0.05, variance analysis was employed to calculate the significance level of the variables. The key and interactive terms of the variables with *p*-values lower than 0.05 were regarded as significant in affecting the model responses, whereas *p*-values greater than 0.05 were regarded insignificant [33,34]. For prediction models, only significant terms are dealt with except those needed to sustain the order of the model.

Face-cantered central composite design (FCCD) with two separate parameters was used to obtain the mix design formulations of the R-ECC. The two separate parameters considered in this investigation were paste aggregate ratio and curing regime. The responses considered in this study were: compressive strength, flexural strength and splitting tensile strength. The software generated 13 mixes with five randomized duplications for each response. The five duplications are the key references the program uses to improve the effectiveness of the experiment towards any possible deviations.

### 2.3. Mix Proportioning of CR-ECC

For the mix proportioning of the CR-ECC, three mixes having different levels of fine aggregate substitution with CR were considered in percentages of 0% (control) to 5%. A constant water binder ratio of 0.32 and a plasticizer of 0.99% by weight of cementitious materials was adopted for each of the mixes. To avoid segregation and bleeding, it was ensured that the amount used for the plasticizer did not exceed the limit permissible for self-consolidating concrete. A constant PVA fibre of 2% by volume fraction was used for elevated temperature investigation. The mix proportion is presented in Table 3.

### 2.4. Sample Preparation and Testing

The test samples were prepared from carefully well mixed quantities of the materials as determined from the mix design. The operation started by dry mixing the OPC, fly ash, CR, and the fine aggregate in a Hobart mixer. The ingredients were mixed dry continuously for 2 min to ensure that they are uniformly dispersed. Water mixed with plasticizer was then added to the dry mixture in the machine and allowed to run for 5 to 8 min more. PVA fiber was finally added, and the mixer was allowed to run for five more minutes to ensure uniform dispersion of the fiber within the mix.

Cubes specimens of 50 mm were used for the compressive strength test and weight loss due to acid attack. For weight loss the cubes were immersed in a 5% sulphuric acid (H_2_SO_4_) solution, and their weight loss was recorded weekly. For change in length, bar (prism) samples of (285 mm × 25 mm × 25 mm) were prepared and immersed in a 5% sodium sulphate (Na_2_SO_4_) solution. The change in length of the samples was monitored every week. Lastly, a non-quantitative efflorescence test was carried out with 2 brick samples (215 mm × 102.5 mm × 65 mm) where one sample was partially immersed in distilled water at a depth of 25.4 mm (1 inch), while the other sample was placed in a drying room without contact with water. The samples were removed after 7 days and left to dry for 2 days. The observation was made on the efflorescence condition on the surfaces of the samples.

For elevated temperature investigation, three different mixtures were selected, which are M6, M3, and M4. These mixtures have a uniform percentage of PVA fibre of 2% while the amount of crumb rubber varies from 0–5%. The samples produced from the three different mixes were tested for compressive strength after being subjected to higher temperatures. The temperatures considered were 24 °C (room temperature as the control); then 100 to 1000 °C at a constant interval of 100 °C (i.e., 100 °C, 200 °C, 300 °C, 400 °C, 500 °C, 600 °C, 700 °C, 800 °C, 900 °C, 1000 °C). After curing the samples for 28 days, they were put in the oven for 24 h at 60 °C to expel any trapped moisture in the matrix. For each elevated temperature considered, the samples were put in a furnace with 1200 °C heating capacity and heated to the desired temperature at the rate of 15 °C/minute. When the required temperature was reached, the heat was maintained at that temperature for one hour to attain thermal stability. The furnace was then turned off and the samples were allowed to cool down naturally to room temperature before subjecting them to compressive strength test in accordance with ASTM C109 [35].

## 3. Result and Discussion

### 3.1. Compressive Strength

**Figure 2:**
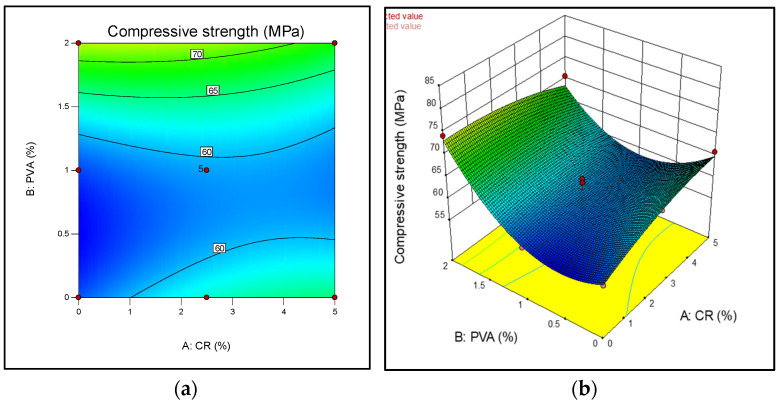
Response surface diagrams for compressive strength. (**a**) 2D contour plot; (**b**) 3D surface diagram.

Figure 2 shows the contour diagram and the 3D response surface diagram for the compressive strength of CR-ECC at 28-days. Compressive strength increases when crumb rubber is used as partial replacement to fine aggregate without the addition of PVA fibers, but there is a small increase in compressive strength when the percentage of crumb rubber content is increased from 0 to 2.5% while the PVA content is more than 0 and less than 1%. By virtue of the inclined contour lines of the 2D diagram, the degree of interaction between the amount of CR and PVA is not much. From the diagrams, it can be observed that the optimum amount of the CR and PVA fiber to obtain the highest compressive strength is at 2.5% and 2% CR and PVA fiber, respectively. As shown in Figure 2, the compressive strength of the crumb rubber ECC ranges between 30 to 75 MPa, which corresponds to previous studies [36,37]. Generally, the addition of crumb rubber into ECC will result in a decrease in compressive strength at 28 days. Various reasons resulted in a reduction in strength. One of the reasons is due to the low compatibility of strain between the soft rubber particles and the hardened cement paste, which may cause a generation of stress at the interface that is characterized by low compressive strength. This agrees with findings from previous studies [38]. Also, the addition of CR increased the amount of air in the matrix, which will ultimately affect the strength [3,38]. The most obvious exception to this trend is trial mix seven which is considered as the control mix. The control mix contained 0% CR and 0% PVA which showed a relatively lower strength at 28 days compared the other mixes that incorporated CR and PVA.

### 3.2. Residual Compressive Strength at Elevated Temperature

**Figure 3:**
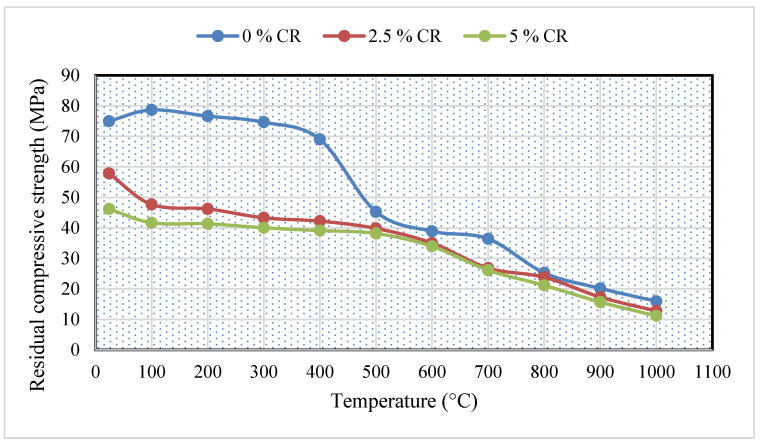
Residual compressive strength of CR-ECC.

In Figure 3, the effect of temperature on CR-ECC is presented. The strength of the samples was generally noticed to reduce with an increase in temperature. The only exceptions are samples with 0% CR at 100 °C. The strength gain, in this case, is associated with the continuous hydration of partly reacted cement and the pozzolanic reaction of fly ash, which was facilitated by autoclaving (heat healing effect) [39]. Heat curing creates a pore-filling mechanism through the action of the pozzolana-derived carbon-silica-hydrate (C-S-H) phase, as reported by Helmi et al. [40]. At around 90–100 °C, heat curing occurs, which is responsible for accelerating the hydration process, thereby modifying both the microstructure and the microchemistry of the concrete [40].

It was noticed that for all the mixes considered (0%, 2.5%, and 5% CR), the addition of CR led to a general downward trend in the compressive strength of the CR-ECC. The strength loss is much more pronounced at higher levels of CR (2.5% and 5%). This can be attributed to the melting of the crumb rubber, creating pores within the matrix thereby reducing the strength by generating internal stresses when the load is applied. As the amount of the CR increases, the number of such voids increases, leading to more reduction in strength. Also, due to the non-polar behavior of the CR particles, water gets repelled from the CR surface while air bubbles get trapped, which leads to the fragile bond between the CR particles and cement paste [3]. Furthermore, these air bubbles also create tiny voids within the CR-ECC matrix resulting in a decrease in strength upon application of load. Hence the higher the amount of CR in the mix, the lower the compressive strength, as can be seen in Figure 3.

At 400 to 500 °C, a sudden drop by 34% was observed for residual compressive strength of CR-ECC incorporating 0% CR. This is a typical behavior of ECC at elevated temperatures. As explained by Wu and Li [41], two mechanisms are responsible for such behavior. The first mechanism is that at a moderate temperature of 200–300 °C, the PVA fibers might have melted resulting in the creation of interconnected channels to relieve the internal pressure. The second mechanism is that these interconnected pore channels became points of weakness upon the application of load. As the temperature became higher than 300 °C, more PVA fiber melted, creating more pore networks that become points of crack propagation upon the application of load. And this resulted in a dramatic decline in the compressive strength at 400 °C. For 2.5% and 5% CR samples, the compressive strength decreases at 100 to 500 °C was very minimal. For the 2.5% CR mix, the residual compressive strength at 100 °C (47.66 MPa) reduced to about 83% of its value at 500 °C (39.81 MPa). Similarly, for 5% CR, the residual strength reduced to only 91% of its value from 100 to 500 °C. On the other hand, the compressive strength for both 2.5% and 5% CR samples decreased dramatically at 600 to 1000 °C. The residual strength at higher temperature (1000 °C) reduced to approximately 11 MPa compared to its value (47.66 MPa for 2.5% CR and 41.7 MPa for 5% CR) at 100 °C.

At 800 °C, all the three different mixes had strength within the acceptable structural concrete strength class [42]. However, at 900 °C, only the control mix (0% CR) had a strength of 20 N/mm^2^ while the remaining mixtures had strengths below the prescribed structural concrete class. At 1000 °C, none of the mixes had attained the acceptable minimum strength required for structural concrete, as the strength reduced to about 20% of the strength at room temperature.

**Figure 4:**
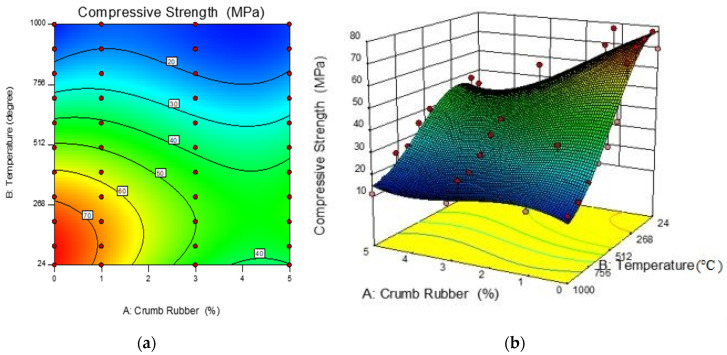
Response surface diagrams for the residual compressive strength of CR-ECC. (**a**) 2D contour plot; (**b**) 3D surface diagram.

The effect of the addition of CR into the ECC mixture, temperature, and the compressive strength can also be presented using three-dimensional surface diagrams. Figure 4 shows the 2D and 3D response surface plots. As shown in the 2D contour plot and 3D surface plot, the inclusion of CR generally reduces the rate of concrete strength loss of the CR-ECC and the trend is more obvious for the elevated temperatures between 500 °C and 1000 °C. It should be noted that the increase of CR content from 1% to 3% just results in a slight decrease in the rate of CR-ECC strength loss after exposure to elevated temperature at 100 to 500 °C. It also be observed that the CR-ECC mixes suffered the highest loss in compressive strength in the temperature range of 600–1000 °C. The optimum compressive strength achieved at 0% of crumb rubber, which is shown in the reddish region in the 2D contour plot in Figure 4.

### 3.3. Cracking and Spalling Behavior

**Figure 5:**
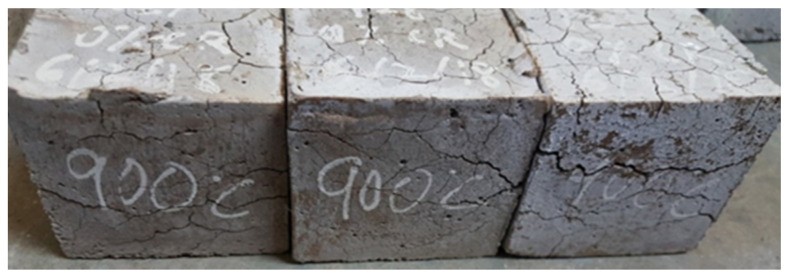
CR-ECC with 0% CR at 900 °C.

**Figure 6:**
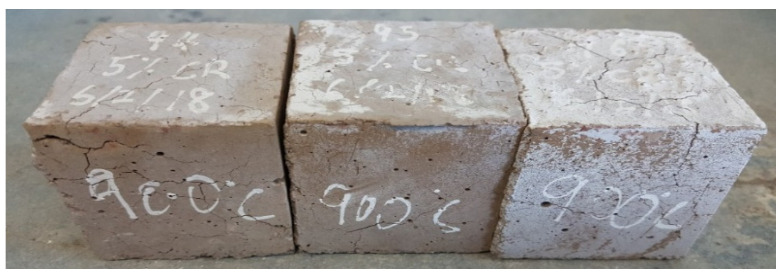
CR-ECC with 5% CR at 900 °C.

Cracking refers to a visible type of concrete defect that has a substantial negative effect on the mechanical and durability properties of concrete [43]. In earlier research conducted on the effect of elevated temperature on concrete, it was reported that at 600 °C all concrete tested were found to suffer some form of deterioration, with only a small portion of their strength left, usually from 7% to 25% for all samples [44]. In this research, however, visible signs of deterioration began to manifest only at temperatures above 600 °C. This agrees with some previously reported works on the behavior of ECC at elevated temperatures [14,15,16]. The presence of CR and PVA fiber helped reduced the development of cracks in the CR-ECC samples. Usually, at high temperatures, the water present within the dense ECC matrix turns into vapor resulting in a buildup of pressure due to lack of escape routes. Consequently, cracks and subsequent spalling of the sample occur. In most cases, when the temperature becomes higher, the spalling is explosive [18,19]. At 170 °C and 230 °C, the CR and PVA fiber, respectively, melted, creating pathways for the water vapor to escape, thereby alleviating the development and propagation of cracks in the CR-ECC at elevated temperatures. It was observed that at 900 °C (Figure 5 and Figure 6), the higher the volume of CR, the lower the cracks on the CR-ECC. The samples having 0% of CR experienced the most cracks as compared to samples having a higher volume of CR. This shows that the CR reduces the initiation and propagation of cracks and prevents explosive spalling as none was noticed in all the mixtures even at a temperature of 1000 °C.

#### Visual Inspection of CR-ECC Specimens after Heating

**Figure 7:**
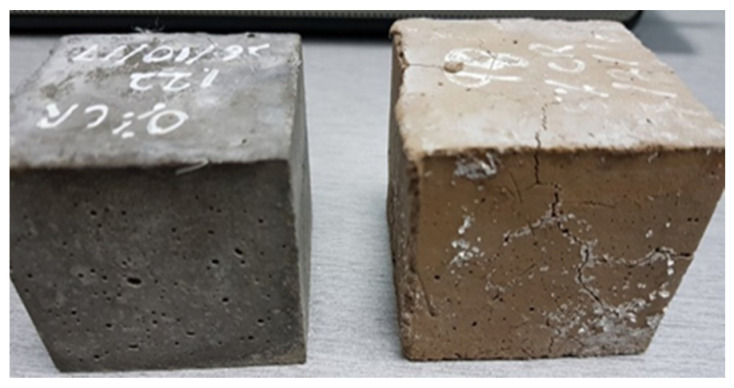
CR-ECC change in color from grey at 24 °C to buff at 1000 °C.

There was a distinct change in color as the samples were subjected to heating at elevated temperatures. As presented in Figure 7, the samples at 24 °C were grey in color, but after heating to the maximum temperature of 1000 °C, the color changed to dark brown or buff. The color change was due to chemical and physical changes that take place in the materials during the heating process [45], which results principally from the gradual water removal and dehydration of the cement paste. Heating the CR-ECC paste to temperature above 500 °C produces several forms of calcium silicates, in general highly porous and weak. Moreover, a 1.5 to 1.0 tobermorite gel, the calcium silicate hydrate, can form and also other silicates such as xonolite and hillebrandite may be formed [46].

### 3.4. Weight Loss Due to Acid Attack

**Figure 8:**
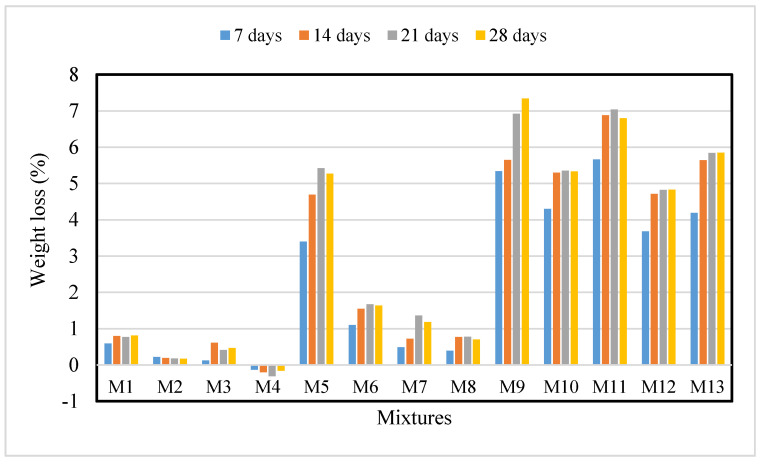
Weight loss vs. exposure time.

Measurement of weight loss of each sample was done weekly for a period of one (1) month and the results are shown in Figure 8. It can be noted that trial mixes M5, M9, M10, M11, M12, and M13 suffered relatively greater weight loss compared to other trial mixes. ECC is vulnerable to acid attack due to its alkaline nature. The reaction between calcareous materials and acid will form calcium salts. The salts cause a reduction of ECC density and cohesion of cement paste. Besides that, calcium-silicate-hydrates (C-S-H) gels react with sulphuric acid to form a fragile gel, thus reducing the strength of the ECC. Furthermore, the formation of soluble calcium salts (i.e., calcium sulphate) will lead to the formation of by-products such as ettringite and gypsum. In the presence of these by-products, the density of the ECC increased and accelerated the hydration process of ECC mortar. Ettringite and gypsum further reduced the permeability of the ECC. In other words, ECC experiences slight weight gain when exposed to an acidic environment [47]. Equations (3) and (4) represent the reaction involved when ECC is exposed to a sulphuric acid solution.

(3) $$Ca{\left(OH\right)}_{2}+H_{2}SO_{4}\to CaSO_{4}\cdot 2H_{2}O$$

(4) $$3CaO\cdot 2SiO_{2}\cdot 3H_{2}O+H_{2}SO_{4}\to CaSO_{4}\cdot 2H_{2}O+Si{\left(OH\right)}_{4}$$

**Figure 9:**
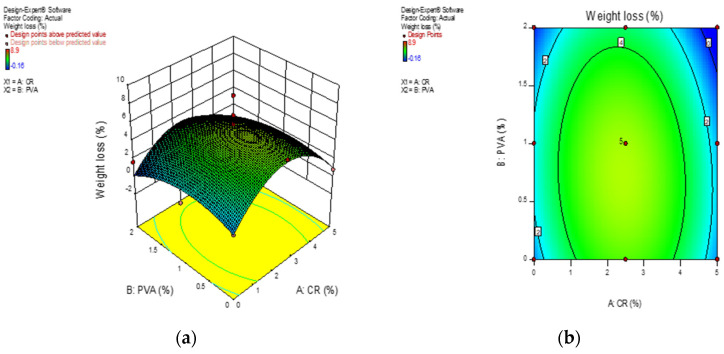
Response surface diagram for weight loss due to acid attack. (**a**) 3D surface diagram; (**b**) 2D contour plot.

Figure 9 shows the 3D response surface diagrams and contour diagrams of percentage weight loss of ECC at 28 days. It can be noted that ECC mixed with 2.5% crumb rubber and 1% of PVA fibers suffered the greatest loss in weight. Looking at the concentric nature of the 2D contour diagram, there is good interaction between the two variables.

### 3.5. Change in Length Due to Sulphate Attack

**Figure 10:**
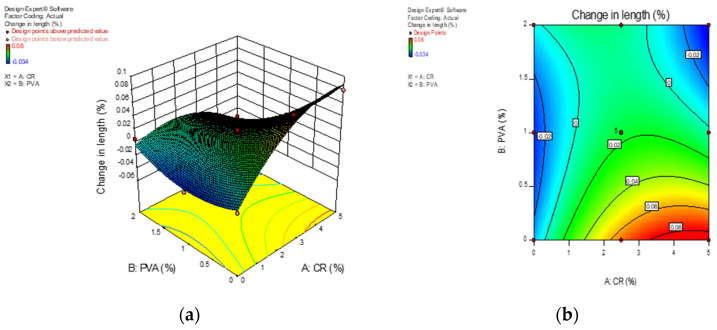
Response surface diagram for change in length due to sulphate attack. (**a**) 3D surface diagram; (**b**) 2D contour plot.

Figure 10 shows the 3D response surface diagram and contour diagram for 28-days change in length (expansion) of ECC in percentage. It can be noted that increasing PVA fiber will result in the change in length of the specimens however, the percentage change in length was reduced by the inclusion of crumb rubber particles, since the deformable rubber could release the internal stress that caused by alkali-silica reaction (ASR). But, the drying shrinkage of the ECC mortar mix was increased when compared with control samples due to the chemical reaction between sulphates and cement paste. Sulphate ions and cement hydration products undergo chemical interaction to yield ettringite and gypsum. These expansive compounds typically lead to loss of mass and strength and increase the volume of ECC. Besides, decalcification of the main cementitious compound, calcium-silicate-hydrates (C-S-H) gels will further deteriorate the condition of ECC. Ultimately, the presence of ettringite and gypsum within the ECC microstructure will result in cracking and spalling of ECC and loss of mechanical strength and its density. On the other hand, several specimens experienced a reduction in length. This is due to the rapid hydration process that occurred which densify the ECC. Furthermore, the hydration process and the presence of CR reduce the permeability and porosity by filling up voids within the ECC. This prevents the penetration of sulphate ions into the ECC to react with the cement hydration products. This agrees with previous research on the effect of sulphate on cement and concrete [47,48].

### 3.6. Anova Analysis of the Developed Models Using RSM

The adequacy of the RSM procedure was tested using analysis of variance (ANOVA), and the result of the statistical test is shown in Table 4. The significance level of each variable and the responses are evaluated using the 95% confidence interval, which corresponds to probability *p*-value ˂ 0.05. In this model, the F-value is 24.41 and it implies that the model is significant. The *p*-value is less than 0.001, which is less than 0.05, indicating that the model terms are significant. There is only a 0.01% chance that an F-value of this magnitude could occur owing to noise. The ANOVA summary shows that the crumb rubber and temperature are significant in affecting the compressive strength with the *p*-value less than 0.05. For this particular situation, model terms B, AB, B^2^, are significant in the model terms while model terms A and A^2^ were found to be insignificant in affecting the compressive strength model. Similar observations were also found in the other response models (weight loss due to acid attack and change in length).

Using the analysis of variance (ANOVA) method, the response surface models developed were validated for the three responses (compressive strength at 28 days, acid attack, and sulphate attack) with the variables as amounts of CR and PVA fiber. The values for the analysis are shown in Table 4. It is observed that PVA fiber and the model are the significant terms in the compressive strength model, while crumb rubber is a non-significant variable. For the weight loss due to acid attack model, the model is significant, although both variable terms are not significant. On the other hand, the variable terms and the model for the compressive strength are significant having a value of more than 95% for the probability. Hence, this indicates the strength of the developed models and that they can be used to get brilliant results in terms of the responses.

The empirical model developed with regard to the actual factors for the compressive strength, weight loss, and change in length of the CR-ECC mixtures, including all the terms, are shown in Equations (5)–(7), respectively. The positive and negative symbols show the antagonistic or synergistic influence of the parameters on the independent responses (strengths). The model equation was developed by ANOVA in which 28 days’ acid attack resistance of ECC can be predicted by substituting the percentages of crumb rubber (CR) and PVA fibers (PVA) respectively.

(5) $$\begin{array}{c} \mathrm{Compressive}\;\mathrm{strength}\;\left(\mathrm{MPa}\right)\\ \;\;\;\;\;\;\;=57.444+2.78016\;\mathrm{CR}-8.54238\;\mathrm{PVA}-1.23\;\left(\mathrm{CR}*\mathrm{PVA}\right)\\ \;\;\;\;\;\;\;-0.25438\;{\left(\mathrm{CR}\right)}^{2}+8.2057\;{\left(\mathrm{PVA}\right)}^{2} \end{array}$$

(6) $$\begin{array}{cc} \mathrm{Weight}\;\mathrm{loss}\;\mathrm{in}\;\mathrm{acid} & \left(\%\right)\\ = & +1.1891+3.1873\mathrm{CR}+2.5932\;\mathrm{CR}-0.143\;\left(\mathrm{CR}*\mathrm{PVA}\right)\\ - & 0.6449\;{\left(\mathrm{CR}\right)}^{2}-1.5653\;{\left(\mathrm{PVA}\right)}^{2} \end{array}$$

(7) $$\begin{array}{cc} \mathrm{Change}\;\mathrm{in}\;\mathrm{length} & \left(\%\right)\\ & =-0.0133+0.0439\;\mathrm{CR}-0.0361\;\mathrm{PVA}-0.0138\;\left(\mathrm{CR}*\mathrm{PVA}\right)\\ & -0.0047\;{\left(\mathrm{CR}\right)}^{2}-0.0205\;{\left(\mathrm{PVA}\right)}^{2} \end{array}$$

**Figure 11:**
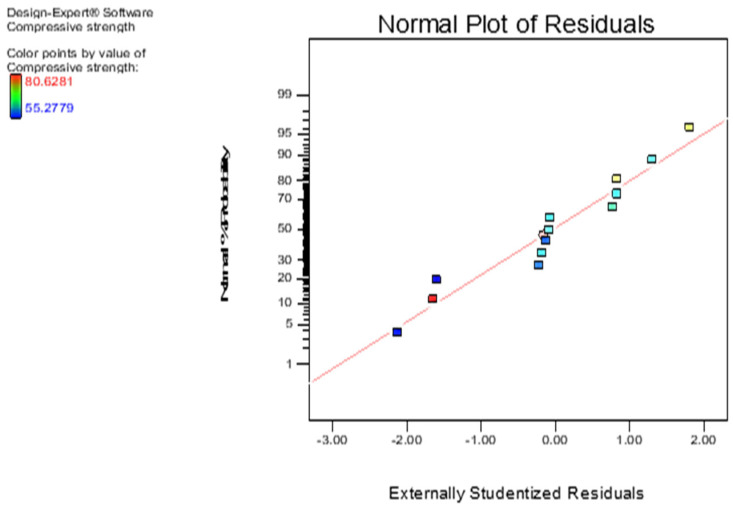
Normal plot of residuals of compressive strength.

From Table 5, the difference between adjustable R squared values and the predicted values for all the models is below 0.2, and they have a probability value of more than 95%. Hence, there is a good agreement between the predicted R-squared and the adjustable R-squared values. Further, the 28-compressive strength, acid attack, and sulphate attack models can be respectively validated using the normality plots of residuals depicted in Figure 11. By virtue of the position of the points along the normality line, it can be deduced that the models give an accurate result as expected. 

**Figure 12:**
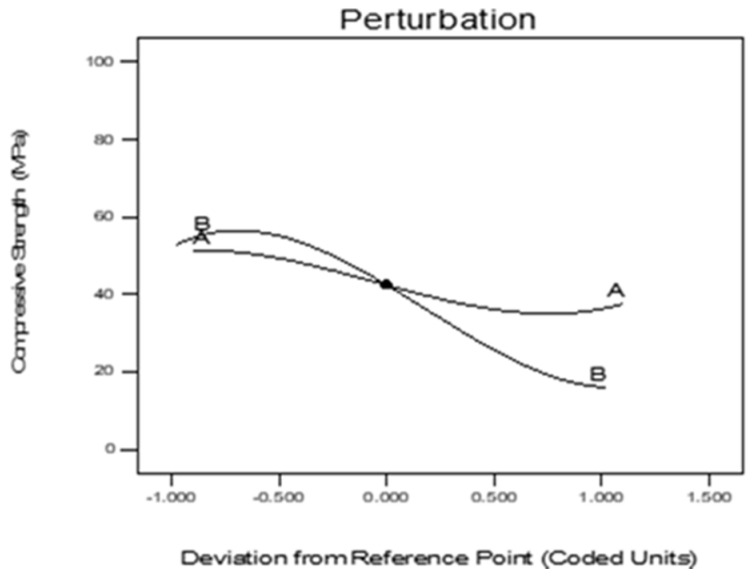
Perturbation plot of compressive strength.

Figure 12 illustrates the perturbation of the measured properties of the CR-ECC, CR, and elevated temperature. The plot showed the comparative effect of the CR and elevated temperature, which can enhance the strength. The interaction lines represented the sensitivity of the response. From Figure 12, the y-axis represents the residuals, while the x-axis is the fitted values. The residuals are scattered around the origin. This shows that the linear relationship assumption is reasonable. For the residuals to concentrate along the horizontal region (horizontal band) at the origin line, this suggests that the variances of the error terms are equal. No single residual value stands out from the basic random pattern of residuals. This indicates the absence of outliers.

## 4. Conclusions

This paper experimentally investigated the effect of elevated temperature on the compressive strength and durability properties of the crumb rubber engineered cementitious composite. The following conclusions were drawn from this investigation:

- The addition of CR in ECC reduces the occurrence of cracks and explosive spalling at elevated temperatures. It was realized that samples with higher CR percentage developed less cracking at high temperatures.
- The compressive strength of CR-ECC reduces with an increase in CR addition and a rise in temperature. However, at a temperature of 100°C, there was an increase in compressive strength at 0% and 1% CR addition that is attributed to the heat curing effect.
- All the mixes considered (0%, 2.5%, and 5% CR) attained the compressive strength requirement for structural concrete at elevated temperatures up to 800 °C.
- CR-ECC experiences slight weight gain and a reduction in strength when exposed to an acidic environment.
- Most CR-ECC samples experienced a reduction in length when exposed to sulphate because due to the rapid hydration process, there was a reduction in permeability and porosity of the CR-ECC which prevented the penetration of the sulphate ions whose reaction products cause the expansions.
- RSM models were developed to predict the compressive strength, acid resistance, and sulphate resistance at 28 days based on the percentages of PVA fibers and crumb rubber. The difference between adjusted R-squared and predicted R-squared for each model is less than 0.2, and they possess at least a 95% confidence level.

## Figures and Tables

**Table 1:** Oxides and properties of OPC and FA.

Oxide Composition (%)	OPC	FA
SiO_2_	20.76	57.01
Al_2_O_3_	5.54	20.96
Fe_2_O_3_	3.35	4.15
CaO	61.4	9.79
MgO	2.48	1.75
MnO	−	0.033
K_2_O	0.78	1.53
Na_2_O	0.19	2.23
LOI	2.2	1.25
specific gravity	3.15	2.38
Blaine fineness (m^2^/kg)	325	290

**Table 2:** Properties of PVA fiber.

Type	Grade of Fiber	Specific Gravity	Length of Fiber (mm)	Diameter of Fiber (µm)	Aspect Ratio	Tensile Strength (MPa)	Modulus of Elasticity (GPa)
PVA	REC 5–15	1.3	12	40	462	1600	41

**Table 3:** Mixture proportion of CR-ECC.

Mix	CR (%)	PVA Fibre (%)	Crumb Rubber, (kg/m^3^)	River Sand, (kg/m^3^)	Fly Ash, (kg/m^3^)	Water, (kg/m^3^)	OPC, (kg/m^3^)	SP^x^ (kg/m^3^)
1	5	0	23.35	443.65	700	187	583	19.89
2	5	1	23.35	443.65	700	187	583	20.53
3	2.5	2	11.68	455.32	700	187	583	20.53
4	5	2	23.35	443.65	700	187	583	19.89
5	2.5	1	11.68	455.32	700	187	583	19.89
6	0	2	0	467	700	187	583	20.53
7	0	0	0	467	700	187	583	16.68
8	0	1	0	467	700	187	583	16.68
9	2.5	1	11.68	455.32	700	187	583	19.89
10	2.5	0	11.68	455.32	700	187	583	19.25
11	2.5	1	11.68	455.32	700	187	583	19.89
12	2.5	1	11.68	455.32	700	187	583	19.89
13	2.5	1	11.68	455.32	700	187	583	19.89

SP^x^: Superplasticizer.

**Table 4:** Summary of variance analysis.

Response	Source	Sum of Squares	df	Mean Square	F-Value	*p*-Value	Remarks
Compressive Strength (MPa)	Model	684.71	5	136.94	24.41	0.0003	significant
	A-CR	3.13	1	3.13	0.56	0.4792	insignificant
	B-PVA	148.79	1	148.79	26.53	0.0013	significant
	AB	37.82	1	37.82	6.74	0.0356	significant
	A^2^	12.21	1	12.21	2.18	0.1836	insignificant
	B^2^	325.37	1	325.37	58	0.0001	significant
Weight loss (%)	Model	82.46	5	16.49	4.14	0.0454	significant
	A-CR	1.22	1	1.22	0.30	0.5980	insignificant
	B-PVA	4.81	1	4.81	1.21	0.3084	insignificant
	AB	0.51	1	0.51	0.13	0.7307	insignificant
	A^2^	44.86	1	44.86	11.26	0.0122	significant
	B^2^	6.77	1	6.77	1.70	0.2337	significant
Change in length (%)	Model	0.014	5	0.0029	29.56	0.0001	significant
	A-CR	0.0016	1	0.0016	16.52	0.0048	significant
	B-PVA	0.0053	1	0.0053	54.49	0.0002	significant
	AB	0.0048	1	0.0048	49.13	0.0002	significant
	A^2^	0.0024	1	0.0024	24.83	0.0016	significant
	B^2^	0.0012	1	0.0012	11.96	0.0106	significant

**Table 5:** Validation by model terms.

Response/Model Terms	Compressive Strength	Weight Loss	Change
Standard deviation	2.37	0.72	0.005
Mean	63.13	−3.11	0.0084
C.V.%	3.75	23.24	59.48
PRESS	179.64	20.16	0.001
R-Squared	0.9458	0.9528	0.9699
Adjusted R-Squared	0.907	0.9191	0.9483
Predicted R-Squared	0.7519	0.7399	0.8324
